# Supplementary material for: Early Circulating Edema Factor in Inhalational Anthrax Infection: Does It Matter?
Source: Microorganisms. 2024 Jan 31;12(2):308. doi: 10.3390/microorganisms12020308 (PMC10891819; doi:10.3390/microorganisms12020308)
Supplement: Supplementary file 1 [file microorganisms-12-00308-s001.zip › Figure S1.pdf]

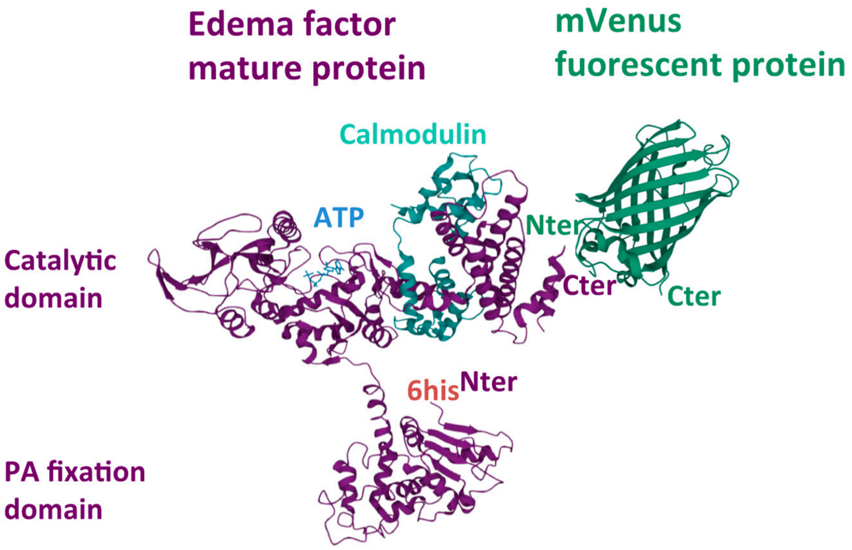

Figure S1. Schematic representation of the EFvenus fusion protein. The edema factor (89 kDa) and mVenus protein (27 kDa) are respectively colored in purple and green (as their N-terminal and C-terminal end). The poly-histidine tag (6his) is indicated in red. The ATP (blue) and calmodulin (turquoise) required for the enzymatic activity of EF are located in their respective site of fixation. 3D protein models are from Protein Database : edema factor (PDB:1XFV), mVenus (PDB:7PNN).
