# Supplementary material for: Early Circulating Edema Factor in Inhalational Anthrax Infection: Does It Matter?
Source: Microorganisms. 2024 Jan 31;12(2):308. doi: 10.3390/microorganisms12020308 (PMC10891819; doi:10.3390/microorganisms12020308)
Supplement: Supplementary file 1 [file microorganisms-12-00308-s001.zip › Figure S2.pdf]

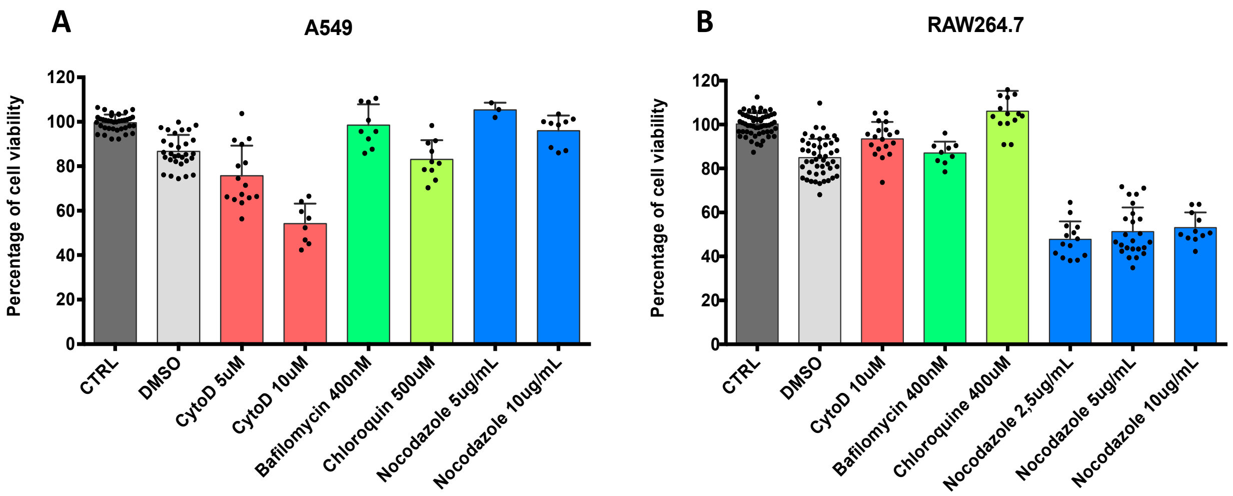

Figure S2. Viability of cell lines exposed to inhibitors. A549 (A) or RAW264.7 (B) cells were exposed to different concentrations of inhibitors for 90 minutes at 37°C, then washed to eliminate non adherent cells. Viability was estimated by cristal violet staining and percentage reported to untreated cells (CTRL). Influence of the vehicle in which inhibitors were reuspended (DMSO) was also integrated.
