# Supplementary material for: Early Circulating Edema Factor in Inhalational Anthrax Infection: Does It Matter?
Source: Microorganisms. 2024 Jan 31;12(2):308. doi: 10.3390/microorganisms12020308 (PMC10891819; doi:10.3390/microorganisms12020308)
Supplement: Supplementary file 1 [file microorganisms-12-00308-s001.zip › Figure S3.pdf]

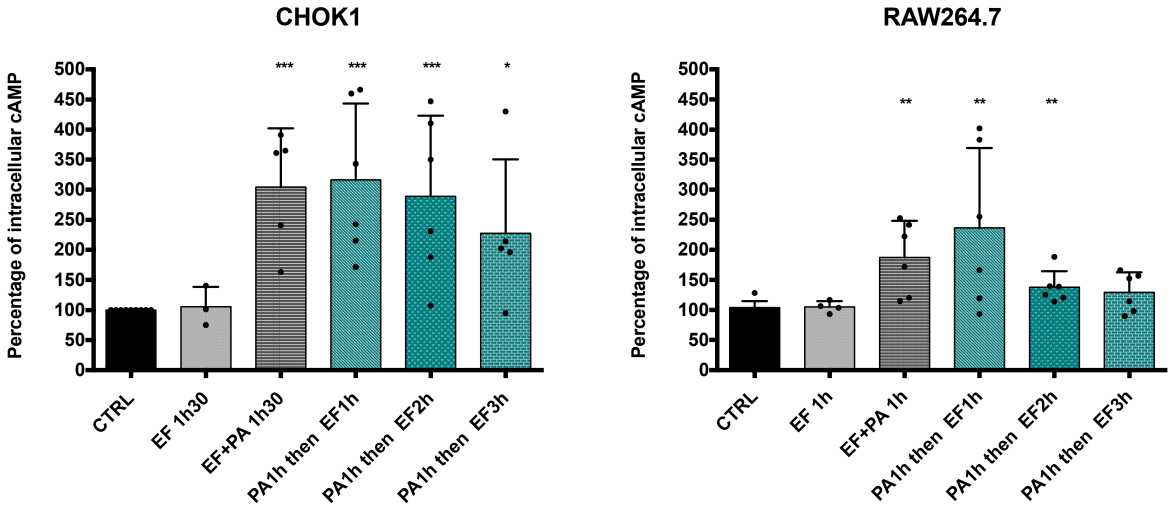

Figure S3. cAMP levels increase after the sequential entry of PA and EF. MDCK, CHOK1, and RAW264.7 cells were incubated with PA (300 nM) for 1 h at 37°C and then washed and incubated again for 1, 2, or 3 h with EF (100 nM). The level of intracellular cAMP was monitored by ELISA and compared to that of cells treated with 100 nM EF ± 300 nM PA after 90 min of exposition at 37°C. Results are shown as the mean with the SD (n ≥ 3). Each dot refers to a single experiment. Values were normalized to control as 100% of the cAMP level. Stars indicate a significative difference between the control (CTRL, untreated cells) and one of the conditions of cell intoxication (nonparametric Mann-Whitney test; *$p < 0.05$, **$p < 0.01$, ***$p < 0.001$).
