# Supplementary material for: Early Circulating Edema Factor in Inhalational Anthrax Infection: Does It Matter?
Source: Microorganisms. 2024 Jan 31;12(2):308. doi: 10.3390/microorganisms12020308 (PMC10891819; doi:10.3390/microorganisms12020308)
Supplement: Supplementary file 1 [file microorganisms-12-00308-s001.zip › Figure S4.pdf]

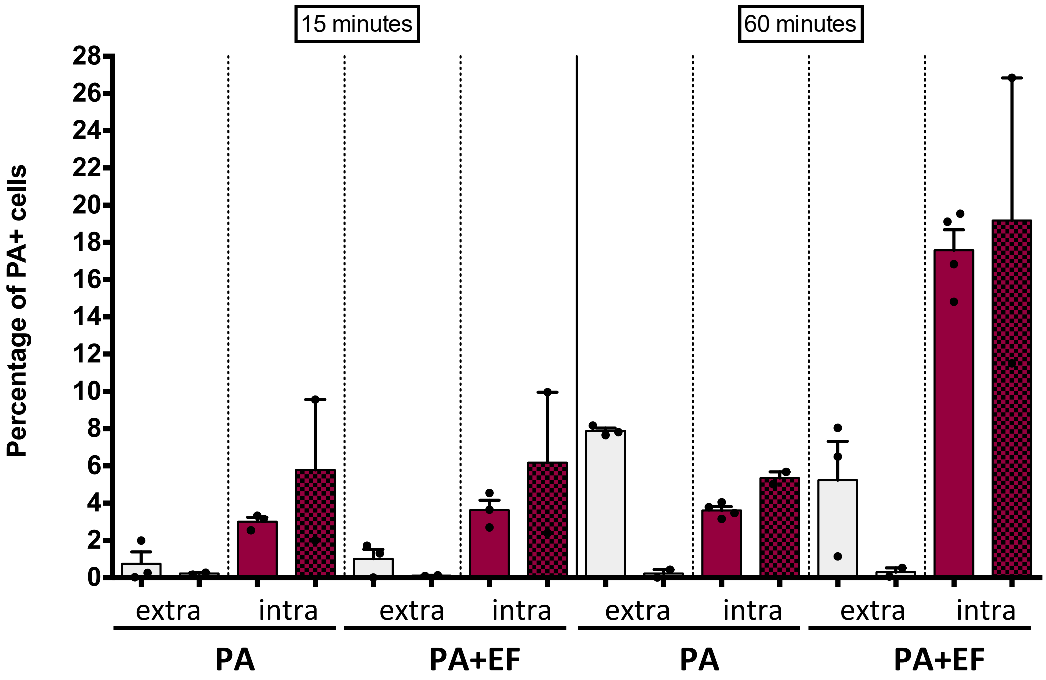

Figure S4. Cell washes remove PA from cell surface of CHOK1. CHOK1 cells were exposed to PA 300 nM with or without EF (100 nM) for 15 or 60 minutes at 37°C. Cells were then washed 1 time (clear bars) or 2 times (motif bars) with PBS before being analyzed by flow cytometry using an biotinylated antibody angainst PA. Using non permabilization condition, the presence of PA at cell surface was quantified (extra, grey bars), and permeabilization allowed access total content including intracellular PA (intra, red bars). Each experiment was done in duplicate, n=2. Percentage of live cells containing PA is represented on the histogram.
