# Supplementary material for: Early Circulating Edema Factor in Inhalational Anthrax Infection: Does It Matter?
Source: Microorganisms. 2024 Jan 31;12(2):308. doi: 10.3390/microorganisms12020308 (PMC10891819; doi:10.3390/microorganisms12020308)
Supplement: Supplementary file 1 [file microorganisms-12-00308-s001.zip › Video S1.pdf]

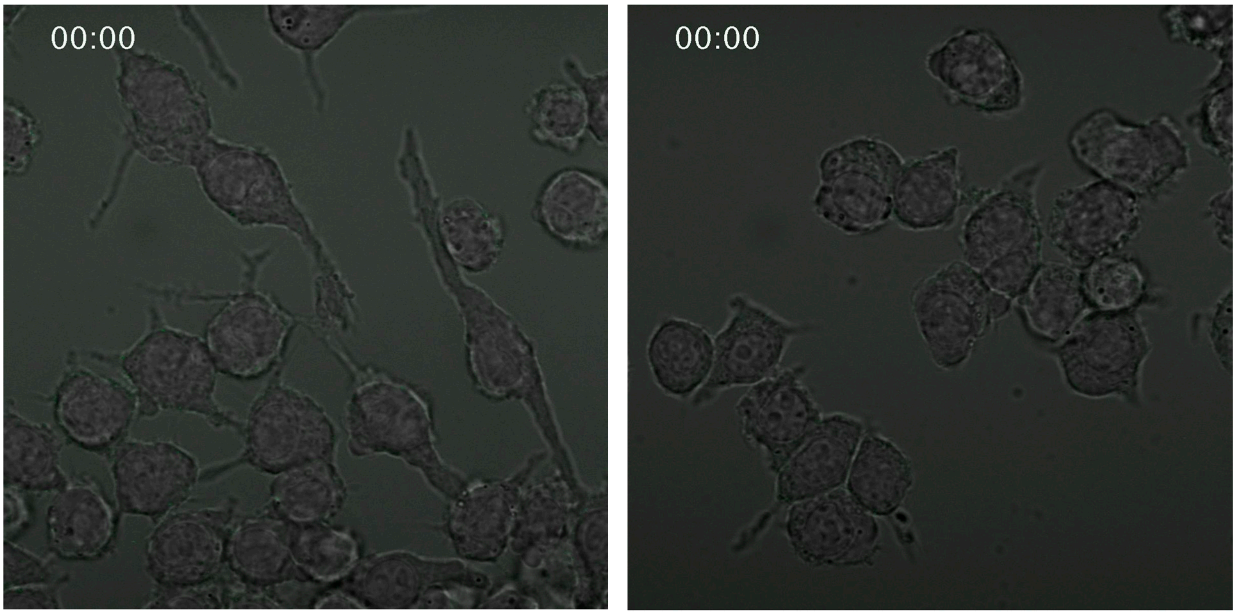

Video S1. Time lapse of EFvenus entry ± PA into RAW264.7 macrophages. RAW264.7 cells were exposed to EFvenus (100 nM) alone (left panel) or in the presence of PA (300 nM) (right panel) and observed by confocal microscopy under live cell imaging conditions. Images were captured every minute, with transillumination and a fluorescence laser for EFvenus (green). The clock at the upper left indicates the time in hours and minutes.
